# Efficacy of slow-release tags impregnated with aggregation-attachment pheromone and deltamethrin for control of *Amblyomma variegatum* on St. Kitts, West Indies

**DOI:** 10.1186/1756-3305-7-182

**Published:** 2014-04-14

**Authors:** Patrick J Kelly, Helene M Lucas, Craig M Randolph, Kate Ackerson, Jason K Blackburn, Michael J Dark

**Affiliations:** 1 School of Veterinary Medicine, Ross University, Basseterre, St. Kitts and Nevis, West Indies; 2 Emerging Pathogens Institute, University of Florida, Gainesville, FL 32611, USA; 3 Department of Geography, University of Florida, Gainesville, FL 32611, USA; 4 Department of Infectious Diseases and Pathology, College of Veterinary Medicine, University of Florida, Gainesville, FL 32611, USA

**Keywords:** Deltamethrin, Slow-release tag, Tick control, *Amblyomma*, Aggregation-attachment pheromone

## Abstract

**Background:**

*Amblyomma variegatum* is an important cause of morbidity, mortality and economic losses in Africa and the West Indies. Attempts to control and/or eradicate the tick from the Caribbean have largely been unsuccessful because of difficulties relating to the biology of the three-host tick and problems with applying acaricides on a regular basis to free-ranging domestic ruminants. While plastic collars impregnated with insecticides are widely and effectively used in companion animals to control external parasites there is little information on this technology in ruminants.

**Methods:**

Over 21 months we tested the efficacy of slow-release plastic tags impregnated with deltamethrin (7%) and aggregation-attachment pheromones (DPITs) in controlling *A. variegatum* on free-ranging cattle on two farms on St. Kitts. The tags were replaced every three months or when found to be lost.

**Results:**

On sentinel animals fitted with tags containing only aggregation-attachment pheromones there were an average of 23.1 ticks per semi-monthly visit although this number varied considerably, peaking in the dry season around May and being lowest in August to October during the wet season. Significantly fewer ticks (3.5 on average) were found on cattle with DPITs at each visit (P < 0.001). Although the DIPTs provided good control (92% on average), they did not significantly reduce *A. variegatum* in the environment with tick numbers on sentinels being higher in the second year of the study, despite up to 44% of animals being fitted with DPITs. The tags were economical, costing 0.2% of the 1% flumethrin pour-on treatment widely recommended for *A. variegatum* control in the Caribbean. The major problem encountered was that 38% of tail tags were lost before they were due for replacement every three months.

**Conclusions:**

Our study has shown that DPITs are cheap to produce, easy to place, only require handling of animals every three months, and are very effective in protecting cattle from *A. variegatum*. Before DPITs can be considered for eradication programs the problems needing to be addressed include loss of tail tags, particularly in thick vegetation, and the optimum number of animals that must be treated to reduce numbers of ticks in the environment.

## Background

The tropical bont tick, *Amblyomma variegatum*, was introduced onto the Caribbean island of Guadeloupe on cattle imported from Senegal in the early 1800s [1]. The tick is well adapted to the climatic conditions in the Caribbean [2] and, by the 1990s, it had spread to 19 Caribbean islands and caused substantial economic loses [3]. One reason for the losses is that *Amblyomma variegatum* has long mouth parts and attachment and feeding results in local infections, abscesses and hide damage. Also, infections with *Dermatophilus congolensis* are associated with the presence of *A. variegatum* and major epidemics of dermatophilosis with up to 90% mortality have been described in the Caribbean [4,5]. Further, *A. variegatum* is the vector of *Ehrlichia ruminantium*, a Gram-negative rickettsia that is the causative agent of heartwater, an acute disease of domestic ruminants causing mortalities of up to 90%. The disease is known to occur on at least three Caribbean islands [6] and might spread to the Americas with disastrous effects on their livestock industries [7]. *Amblyomma variegatum* is also important in human medicine as it is the vector of *Rickettsia africae*, the agent of African tick-bite fever, a spotted fever rickettsiosis affecting local people and tourists in the Caribbean [8].

Because of the economic importance of *A. variegatum* in the Caribbean and possibly the Americas, various attempts have been made by local and international authorities to control and eradicate the tick [9,10]. Local control programs have often been ineffective [11] and apparent eradication of *A. variegatum* from some islands has been short-lived [4,10,12]. There are many reasons for the lack of success including owner non-compliance [4,11], reintroduction of ticks by animal movement or migratory cattle egrets (*Bubulcus ibis*) [13], refugia of ticks on feral livestock or wildlife hosts [14,15], and the difficulties of catching and treating animals kept under free-ranging conditions. Currently, the most common method for control of *A. variegatum* in the Caribbean is the bi-weekly application of 1% flumethrin pour-on acaricide (Bayticol®, Germany). Although effective, it has significant limitations in the Caribbean where livestock are generally kept under free-ranging conditions and are difficult to capture and handle. Also, the procedure is labor intensive and expensive, making regular treatment of all animals unlikely. Intermittent acaricide therapy may lead to the development of resistance and enable *A. variegatum* to persist at low levels that are difficult to detect.

The ideal method for *A. variegatum* control should be cheap, easy to perform, require minimal training, and have a prolonged acaricidal effect to minimize the number of times animals have to be restrained and treated. In the 1990s, slow-release plastic collar and tail tags were developed which deliver acaricides that spread widely over the coat, reaching levels that are toxic for ticks for at least three months [16]. To increase the number of ticks killed, the tags can also be impregnated with aggregation-attachment pheromones that also spread around the hair coat and attract male, female and unfed nymphal *A. variegatum* onto the host over a period of at least three months [16,17]. In a pilot study on Guadeloupe [17], cattle with tags containing the aggregation-attachment pheromone and deltamethrin had significantly fewer *A. variegatum* (80% initially, then 60-70%) than control animals. In a similar study on the closely related species *Amblyomma hebraeum* in Zimbabwe, control varied from poor (50-74%) to excellent (over 95%) using tags containing the aggregation-attachment pheromone and alphacypermethrin and cyfluthrin, respectively [16]. In both studies the cattle were confined to paddocks and only monitored for three months.

To provide more data on the efficacy of slow-release tags under field conditions in the Caribbean and to determine their practicality, we carried out a 21-month study on cattle kept under traditional Caribbean free-ranging conditions. Our findings are reported below.

## Methods

### Study site

St. Kitts is an island in the northeastern Caribbean (17.2°N; 67.1°W) about 2,100 kilometers southeast of Florida. It has an area of 168 square kilometers and consists of the main island and a dry and sparsely populated southeast peninsula. The island has a tropical climate with temperatures varying from 23°C to 31°C and humidity from 63% to 91% [18]. Rainfall averages approximately 1.14 meters/year and is lowest in February-March and peaks in September. The middle of the island is mountainous with peaks reaching 1,300 meters. Between the base of the mountains and the coast there are gently sloping grasslands and abandoned cane fields that are gradually reverting to native bush; these are the primary livestock areas on the island.

Total livestock numbers peaked in the mid-1980s at 22,000 (6,000 cattle, 9,000 sheep, and 7,000 goats), but declined to a total of 2,200 (400 cattle, 800 sheep, and 1,000 goats) in 1990 when *A. variegatum* and dermatophilosis became prevalent [4]. This tick was probably introduced from the sister island of Nevis in 1978 and thereafter spread widely to occupy all areas apart from the central mountainous areas and the dry southeast peninsula. Following the Caribbean Amblyomma Programme, which involved blanket treatment of livestock with biweekly flumethrin 1% pour-on (Bayticol®, Bayer, Germany), St. Kitts was declared provisionally free of the *A. variegatum* in 2001 [10] when livestock numbers had recovered. Subsequent to discontinuation of mandatory flumethrin treatment *A. variegatum* reappeared and became prevalent in all areas, apart from the southern peninsula. Further, outbreaks of dermatophilosis occurred and, although no census data is available, local experience suggested that cattle numbers had decreased substantially when our study began in 2011.

Farms were considered for the study if *A. variegatum* were present; their owners were willing to participate; there were at least 15 cattle present and no other livestock; the cattle could most likely be routinely presented for examination each month.

### Slow-release tags

Two types of slow-release tags were used in the project; one contained aggregation-attachment pheromones alone (PITs) and was used on sentinel animals while the other contained pheromones and 7% deltamethrin (DPITs) (We-Young Industrial and Trading Co., Nanjing, China) and was used on treated animals. The tags (65 x 90 mm) were made from liquid polyvinylchloride (B. F. Goodrich, Avon, OH), plasticizers (Witco Corporation, Avon, OH) and stabilizers (Witco Corporation, Avon, OH) as described previously [16]. The pheromone was formulated from o-nitrophenol, methyl salicylate, 6- dichlorophenol and phenylacetaldehyde (Sigma-Aldrich Chemical Co., St. Louis, MO) in proportions found in natural tick extracts [19,20], mainly 1%, 1%, 0.2% and 0.1% of 100 g PVC, respectively. The tags were made at the University of Florida and immediately sealed in aluminium foil pouches before being shipped to St. Kitts where they were stored at room temperature (around 27°C) until use.

The tail tags were attached to about 100 tail hairs as close to the end of the living tail as possible using cyanoacrylate adhesive (Bondini-2, Pro-Tel Inc, Santa Monica, CA) and about ten heavy duty 1 centimeter metal staples (Staplex, Brooklyn, NY) as described previously [17]. The collar tags were attached to polyurethane coated neck collars (Biothane®, Bio-Plastics, North Ridgeville, OH) using cyanoacrylate adhesive (Bondini-2, Pro-Tel Inc, Santa Monica, CA) and about ten heavy duty 1 centimeter metal staples (Staplex, Brooklyn, NY). As tags have been found to release pheromone and deltamethrin for prolonged periods [17] they were replaced every 3 months, or sooner if they were found to be missing.

While there are no data on the percentage of animals that need to be treated for effective tick control in the herd, previous experiments suggest 10% as a reasonable starting estimate [17]. The tagged animals are expected to assimilate with the free-roaming population and attract and kill *A. variegatum* in the area. Monitoring the population over a roughly two-year period would be required to assume that all adult *A. variegatum* had been removed [12].

### Animals

Approval of the study was obtained from the Institutional Animal Care and Use Committee of Ross University School of Veterinary Medicine and the University of Florida. Cattle used in the study were all adults that could be caught by the farmers for study visits. The animals all had neck collars made from canvas straps to which lengths of chain or rope (around 20 meters) were tied. Before a study visit the animals were restrained by attaching the ends of their chains/ ropes to trees or stout bushes. To immobilize the cattle for inspection, the free chain/rope attached to the collar was progressively taken in as the animal was gently driven towards the tree/bush to which it was attached. When the animal was closely tied to the tree/bush it was sedated and given analgesia for tick removal by injection of xylazine (0.1 to 0.2 mg/kg IM) with a blow dart (Dan-Inject®, Austin, TX; Pneu-Dart®, Williamsport, PA). Following darting, the animals were left undisturbed for around 15 minutes until sufficiently sedated to be handled without stress.

Study visits to the farms were made approximately monthly, depending on availability of the owners and water. Although the cattle were free to roam they generally returned to the farms every day or two for water, which was not readily available on surrounding lands, especially if there was no rain. By withdrawing water for a day before visits the farmers could generally ensure many of the study animals would be available.

### Tick samples

During the approximately monthly study visits the sedated animals were examined thoroughly for *A. variegatum*. All ticks found were removed and stored in 100% ethanol until their identity was confirmed using standard morphological criteria. Control of ticks on animals with DPITs was calculated using the formula:

$$\mathrm{Control}\left(\%\right)=100\times \left(m_{c}–m_{t}\right)/m_{c}$$

where m_c_ is the mean number of live ticks on the control animals with PITs and m_t_ is the mean number of live ticks on animals with DPITs [17].

### Statistical analysis

Statistical analysis was performed using SPSS V.20 (IBM Corp., Armonk, NY). A P ≤ 0.05 was considered significant.

## Results

### Study sites

Two farms were finally selected for the study, one (Farm A; 17°20′25.66″N and 62°43′53.27″W) on the north of the central mountain range and one (Farm B; 17°18′36.10″N and 62°47′14.67″W) on the south. Farm A covered about 12 hectares and was situated at the base of the central mountains on the Atlantic side of the island about 120 m above sea level. It had a deep impassable ravine on the east side and an incomplete 2 meter diamond wire fence around the other sides which did not prevent cattle straying up into the tropical forest of the mountains or down into grasslands and abandoned cane fields towards the coast in search of grazing. Piped water was available at three troughs in the center of the farm when considered necessary by the farmer. The vegetation on the farm consisted of patches of grasses (*Cynodon dactylon* and *Panicum maximum*), acacia scrub, a variety of mature trees, and patches of *Crotalaria pallida, Xanthium stumarium*, and *Phyllanthus amarus*. Farm B was on the Caribbean side of the island midway between the base of the central mountains and the coast, approximately 60 m above sea level. It covered 21 hectares and was unfenced. The vegetation consisted of grassland, predominantly *Cynodon dactylon* and *Panicum maximum*, as well as scattered acacia scrub, *Solanum torvum* and various mature trees.

### Animals

Initially there were 18 mixed breed cattle on Farm A, but over the course of the study three died and one was sold. On Farm B there were 17 mixed breed cattle for the duration. There were no livestock permanently situated on the lands immediately surrounding Farms A and B and no animals strayed onto the farms during the study. Prior to the commencement of the study, tick control on both farms had been with topical applications of 1% flumethrin (Bayticol Pour-on®) when animals were thought to have large tick burdens by the owners. No treatments had been given on the farms for at least two weeks prior to the start of our experiments.

From the outset, dermatophilosis occurred commonly on the cattle, particularly on A, with a total of two deaths and 19 animals requiring one to three treatments. Although there was no obvious correlation between tick numbers or presence of DPITs or PITs on the incidence of dermatophilosis there was a strong belief amongst the farmers that the presence of *A. variegatum* was directly linked to the disease. There was thus strong pressure for more animals to be treated with DPITs. Initially there were two sentinel animals with PITs and two cattle treated with DPITs on each farm but, due to the dermatophilosis, we increased the number of cattle with DPITs to seven on Farm A and five on Farm B by month 10 of the first year of the study. Over the same period, we increased the number of sentinel animals with PITs to four on each farm.

At each visit, variable numbers of animals were secured by the farm owners for examination (Table 1). Data on animals requiring treatment for dermatophilosis were not included in analyses as the effects of the disease on tick numbers could not be determined.

**Table 1:** **Tick collection data on sentinel animals with pheromone impregnated slow-release tags (PITs) and animals treated with 7**% **deltamethrin and pheromone impregnated slow-release tags (DPITs) on farms on the north (A) and south (B) of St. Kitts**

	**Farm A**				**Farm B**			
**Date**	**Average ticks on (N) animals with PITs**	**Average ticks on (N) animals with lost tags**	**Average ticks on (N) animals with DPITs**	**Control**	**Average ticks on (N) animals with PITs**	**Average ticks on (N) animals with lost tags**	**Average ticks on (N) animals with DPITs**	**Control**
**11-Feb**	46(2)				3(2)			
**11-Mar**	22(2)				4(2)			
**11-Apr**	9.5(2)	np*	0(1)	100%	1.5(2)	np	0(2)	100%
**11- May**	107(1)	46.3 (3)	9(2)	92%	28(2)	np	0(2)	100%
**11-Jun**	35(1)	np	12.4(5)	65%	23(2)	0 (1)	0(1)	100%
**11-Jul**	27(1)	np	7.3(3)	73%	9(2)	np	0(2)	100%
**11-Aug**	1.5(2)	np	0(1)	100%				
**11-Sep**								
**11-Oct**	7(2)	13.5 (2)	2.5(2)	64%	3(2)	np	0(2)	100%
**11-Nov**	11(1)	np	0(1)	100%	4.5(2)	0 (1)	0(4)	100%
**11-Dec**	7(5)	8.5 (2)	np		6(2)	5 (1)	0(2)	100%
**12-Jan**	11.75(4)	13.25 (4)	np		12.7(3)	np	3(4)	77%
**12-Feb**					16(4)	14 (1)	0(2)	100%
**12-Mar**	37.5(2)	19.5 (2)	np					
**12-Apr**	49(2)	30 (4)	3(1)	94%	41.7(3)	7.3 (3)	2(2)	95%
**12-May**	101(1)	24 (1)	15(3)	86%				
**12-Jun**	30(1)	19.5 (2)	np		15.5(2)	17 (1)	1(2)	94%
**12-Jul**	16(1)	np	4.3(3)	73%				
**12-Aug**					6.5(4)	5 (2)	0(2)	100%
**12-Sep**								
**12-Oct**	8(1)	np	0(2)	100%	18(1)	1 (1)	0.5(2)	97%

*no animals presented with lost tags.

During each farm visit that was made (32; Farm A 17, Farm B 15), we examined an average of 2.5 (81/32; Farm A 40, Farm B 41) sentinel animals with PITs, 1.5 (48/32; Farm A 20, Farm B 28) animals with DPITs, and 1(33/32; Farm A 22; Farm B 11) animal that had lost its tags before they had been in place for three months.

### Tags

The tags were cheap to produce, costing 0.22 US$ for a PIT and 0.46 US$ for a DPIT, excluding equipment costs. The DPITs could provide three months of protection for approximately 0.2% of the cost of six government subsidized Bayticol® treatments on St. Kitts.

Initially animals were sedated using Pneu-Dart® darts but due to the large number of darts that failed to detonate (up to 50% on a particular day) the simpler and cheaper Dan-Inject® darts were used with almost 100% efficacy. Once the animals were immobilized and sedated, the tags were generally easy to place and no neck collars were lost during the study. Although no tags were lost from the collars, only 62% (60/97) of the tail tags were retained for 3 months. Loss was greatest on Farm A (48%; 24/50), which had considerably denser scrub vegetation than Farm B (28%; 13/47) (χ2 = 3.43, P = 0.064). The tail tags appeared to be lost most frequently towards the end of the three month period (data not shown). There was no predisposition for specific animals to lose tail tags. Examination of lost tail tags indicated this was not due to glue or staple failure. Rather, when tags became trapped in fences or dense foliage, they and the hair to which they were attached were wrenched from the tail.

The purity of the deltamethrin used in the tags was confirmed by the California Animal Health & Food Safety Laboratory System (CAHFSL, Davis, CA) prior to study initiation. Tags tested for deltamethrin when they were removed at the end of the 3 month period on the animals had deltamethrin levels ranging from 4.5-5.9%.

### Ticks

Data on ticks on animals requiring treatment for dermatophilosis were not included in analyses as the effects of the disease on tick numbers could not be determined. From the healthy animals we collected a total of 2,318 *A. variegatum* with the majority (1,527; 66%) being found on sentinel cattle with PITs and only a small percentage (183; 8%) on animals with DPITs that had been placed within the previous 3 months (Table 1). The remaining 25% of ticks (608) came from animals (33) that had lost their tail tag within the previous three months. Overall, we collected substantially more male than female *A. variegatum* with the overall male to female ratio being 2.7:1 (623/1695). This was also the case for the individual farms (Farm A [3.1:1; 1234/399] and Farm B [2.1:1; 461/224]) and for treated (2.1:1; 123/60) and untreated cattle (2.7:1; 1109/418).

There were more ticks collected on Farm A (1633) than Farm B (685) and Farm A had a greater tick pressure with an average of 31.4 ticks per sentinel (973 ticks in 31 sentinel examinations) compared to 15.9 on Farm B (554 ticks in 35 sentinel examinations) (t-test, P = 0.05). Numbers of ticks on sentinel animals on both farms peaked around May and were at their lowest levels in August to October (Table 1). This apparent cycle occurred on both farms in both years of the study. The tick challenge on the farms in the second year of the study was greater with an average of 30.2 ticks/ sentinel (Farm A 433/23 and Farm B 202/21) in year one and an average of 30 ticks/sentinel (Farm A 328/8 and Farm B 264/ 14) in year 2. This difference, however, was not significant (t-test; P = 0.064). The increase in ticks in the second year occurred despite DPITs being used on higher percentages of animals on both farms (Farm A: from 11% (2/18 animals) to 44% (7/16 animals on average); Farm B: from 12% (2/17 animals) to 29% (5/17 on average).

Placing DPITs on cattle greatly reduced infestation with *A. variegatum* on both farms with an average of only 3.5 ticks found on a treated animal at any examination (183/53) compared to an average of 23.1 ticks on a sentinel animal (1527/66) (t-test, P < 0.001). Overall, significantly fewer ticks were found on cattle with DPITs on Farm B (0.7; 19/29) compared to those on Farm A (8.4; 167/20) (t-test, P < 0.001). Cattle that had been treated with DPITs for some of the preceding three months, but had lost their tags before being reexamined, had lower average tick levels than sentinels on both Farm A (24.6; 492/20) and Farm B (10.5; 116/11). These levels, however, were higher than those of animals that received the full effect of the DPIT treatment.

The overall tick control efficacy on cattle with DPITs was 92% but this varied widely from 64 to 100% during any single visit (Table 1). Average tick control was higher on Farm B (97%) than on Farm A (86%) (t-test, P = 0.022).

## Discussion

Our study showed that *A. variegatum* is common on the farms we studied on the north and south sides of St. Kitts and also in the surrounding lands where the animals were free to roam. Local experience suggests the farms we studied were not exceptional and that the tick is a problem wherever livestock are kept throughout the main island. It is of note that in 2001, nine years before our study began, St. Kitts was declared to be provisionally free of *A. variegatum* following a period of mandatory 1% flumethrin topical treatment of all domestic ruminants as part of a national tick eradication project which was supported by the Caribbean Amblyomma Programme [10]. By 2006, however, the tick had reappeared, and the provisionally free status of the island was revoked when 76 *A. variegatum* were found during 4,201 farm inspection visits on St. Kitts [12]. During our study five years later, we found an average of 72 *A. variegatum* per farm visit with an average of 23 ticks on each untreated sentinel animal. The figure for our untreated animals is considerably higher than the mean of three to four per infected animal reported from the English Lesser Antilles, which includes St. Kitts, up until 2006 [12]. It is also higher than that reported previously for the French islands of Guadeloupe (6.1) and Marie Galante (6.7-11.5) which had a widely adopted program of biweekly amitraz treatments [21].

While the reappearance of *A. variegatum* on St. Kitts might be the result of reintroduction of the tick, it seems more likely that the tick was never eliminated. In reviewing the Caribbean Amblyomma Programme, Ahoussou *et al.*[12] pointed out that catching and treating all of the predominately free-ranging livestock on the Caribbean islands was not practical and that there were likely isolated populations of free-ranging animals that were not treated and thus able to harbor and spread *A. variegatum*. This is particularly problematic with *A. variegatum* because it is a three host tick with a long life cycle of six months to over three years [22,23]. The tick can thus survive in isolated areas only occasionally visited by free-ranging livestock and thereby appears to be eradicated when not seen on animals during routine inspections. Further, females lay very large numbers of eggs (around 20 000) with high hatching success rates of about 60% [23], which facilitates reinfestation of pastures.

Resurgence of *A. variegatum* was also promoted by a number of other factors. Firstly, the cessation of mandatory and free flumethrin treatments of livestock that followed the award of provisionally free status in 2001 resulted in administration of fewer acaricide treatments and hence the ability of refugia of *A. variegatum* to expand their range. Secondly, the uneconomical sugar industry on St. Kitts closed in 2005 and the sugar cane fields, which occupied large areas of the island, were abandoned and no longer an illegal site for livestock. The fields therefore became additional grazing areas that enabled livestock to range even more freely than before, making the animals even more difficult to find, monitor and treat for ticks on a regular basis [9]. Further, it expanded the area where *A. variegatum* could survive in the environment and find suitable hosts. Finally, the Caribbean climate is ideally suited to the tick [22], which enables it to survive for long periods and to maximize its reproductive potential.

Our study showed the numbers of *A. variegatum* varied seasonally which is consistent with previous reports. Peak numbers were around May in both years and on both farms which is similar to the situation described in Guadeloupe in 1985 to 1987 [22]. This period corresponds to the start of the rainy season on St. Kitts (WeatherSpark.com). The situation is similar in Africa where adult *A. variegatum* numbers also peak in the rainy season [24], presumably as this ensures conditions of humidity and temperature ideal for survival of the ticks and their progeny.

Our finding that more male than female *A. variegatum* (3.1:1) attached to both treated and sentinel animals is consistent with previous reports of ratios of 4:1 for Guadeloupe in 1992 [25] and 2.3:1 for St. Kitts between 1998 and 2002 [9]. A study in Burkina Faso in Africa, where annual rainfall is similar to the Caribbean but occurs principally in a five-month rainy season, demonstrated relatively low percentages of attached females compared to males (from 10%) during the beginning of the rainy season [26]. Attachment rates of almost equal numbers of males and females occurred later in the rainy season, however, and this was assumed to be because females require greater humidity or number of rainy days before female host-seeking activity is activated. This would prevent engorgement and egg laying when rain was sparse and survival of eggs and larvae would be compromised. It may be that the Caribbean is too dry for maximum attachment rates of females and this limits the population of *A. variegatum* in the region. The females that do attach, however, have normal fertility, which is preserved through sex ratios of 4:1 to 1:4 [22].

In general, the tick challenge in our study (an average of 23 ticks per sentinel per visit) was greater than that reported in the two previous studies on the efficacy of the slow-release tags, which was, on average, 2–15 per animal per week in Zimbabwe [16] and 5–14 per animal per week in Guadeloupe [17]. There was significantly more tick pressure on the farm on the north side of the island. Although this might have been as a result of previously lower tick control or denser vegetation which might be more favorable to survival of *A. variegatum*, it could also be due to weather factors with the south side of the island being somewhat drier as it is in the rain shadow area created by the central mountains. We were unable to collect weather and geographical data, which could have helped resolve the issue.

The difference in tick pressure between the farms could also have been because of resistance to deltamethrin and this could account for the significantly lower average control seen on Farm A (86%) as compared to Farm B (97%). Although resistance to deltamethrin has been reported in ticks [27] there is only reference to deltamethrin resistance in one species of *Amblyomma*, *A. cajennense* in Brazil [28]. In 1991, Garris and Barre [29] reported there was no evidence of resistance in *A. variegatum* from Guadeloupe or Puerto Rico at the time. The tags we tested from both farms A and B after 3 months usage still contained significant deltamethrin levels indicating there were no environmental factors, such as UV damage or exposure to water, which might have led to differential leaching or inactivation of the acaricide between farms and reduced efficacy. Our data suggests there might be resistance to deltamethrin by *A. variegatum* and, if DPITs are considered for further studies, this possibility should be explored.

In common with the previous reports on the slow-release tags [16,17], we found the DPITs provided very good control of *A. variegatum*, significantly reducing their numbers on treated animals and providing an average of 92% control throughout the 21 month study. In a short three month study on Guadeloupe, cattle had 62 to 85% fewer *A. variegatum* after they were treated with DPITs [17]. In Zimbabwe, there was over 92% control of *A. hebraeum* with slow release tags impregnated with cyfluthrin or flumethrin, but only 50-75% with alphacypermethrin [16].

Similar to previous studies, we found the slow-release tags were relatively easy to make, only requiring an oven and standard laboratory equipment (scale, glassware, etc.). They were also economical, costing considerably less than treatments with commercially available pour-on formulations, even when the price of the latter was subsidized by government.

Although the DPITs were very effective in controlling *A. variegatum* on the cattle to which they were attached, there was no evidence they decreased the free-living populations of *A. variegatum* in the environment during the course of the 21 month study (Table 1). Instead of tick numbers falling in the second year of the study, there was rather an increase in ticks on sentinel animals, although this was not statistically significant. This was despite the fact that the percentage of animals with DPITs was increased on both farms in the second half of the project. Further, over 2,000 *A. variegatum* were removed from the population pools during the study when ticks were manually collected from the cattle in the study. Total numbers of *A. variegatum* in an area are particularly susceptible to acaricide use on cattle as 95% of all stages of the tick are found on ungulates, in particular cattle which are far more frequently infested than sheep or goats (25:1) [14]. It has been estimated that, without reinfestation, regular acaricide application to cattle with a stocking density of 2–4 animals per hectare would remove all *A. variegatum* in 3–5 weeks [22]. The failure of DPITs in our study to significantly alter the free-living pool of *A. variegatum* was probably because untreated cattle were free to wander into areas of poor tick control where they acquired ticks that were carried back to the study farms when cattle returned for water. The optimal percentage of animals to be fitted with DPITs to bring about significant reductions in the free-living tick populations needs to be determined but will probably be more than the 29% to 44% used in our study.

The major problem we experienced with the slow-release tags was the relatively high rate of tail tag loss (38%) before they were due for replacement at three months. Similar problems were encountered in a study on pastured cattle in Guadeloupe where 45% of the tail tags were lost and needed replacing during the three month study [17]. Initial loss of tags was 3% per week but this rose to 9% of tags lost each week in the final month of the study. When the tags were applied as described in our study and tested on dairy cattle in Zimbabwe, 15% were lost in the first month [16]. The loss of tags reduces the overall efficacy of tick control and means increased work and expense for farmers to replace them. In all previous reports, and in our study, tail tags were lost because the tag and hair were pulled from the tail rather than due to loss of adhesion of the tags to the tail hair. Methods to ‘streamline’ the tags into the tail are required to prevent them from catching and snagging on vegetation and objects and being wrenched from the tail together with the tail hairs.

## Conclusions

In conclusion, our study has shown that DPITs are cheap to produce, easy to place, and very effective in controlling *A. variegatum* on cattle. They are, however, relatively easily lost from the tails, particularly in thick vegetation, and, when placed on up to 44% of animals, they do not significantly reduce the total environmental load of *A. variegatum*.

## Abbreviations

PITs: Slow-release plastic tags impregnated with aggregation-attachment pheromones; DPITs: Slow-release plastic tags impregnated with deltamethrin (7%) and aggregation-attachment pheromones.

## Competing interests

The authors declare that they have no competing interest.

## Authors’ contributions

PK was involved in the study design, project management on St Kitts, data collection and processing, and he drafted the manuscript. HL was involved in the study design, managed the technical aspects of the project on St Kitts, and was involved in data collection and processing, and manuscript preparation. CR and was involved in project management, data collection and sample processing, and reviewing the manuscript. KA was involved in project management, data collection and sample processing, and reviewing the manuscript. JB was involved in the study design, data processing and drafting the manuscript. MD was involved in the study design, project management in Florida, data processing and drafting of the manuscript. All authors read and approved the final manuscript.
